# The Role of Effector-Specific Task Representations in Voluntary Task Switching

**DOI:** 10.5334/joc.255

**Published:** 2023-01-13

**Authors:** Victor Mittelstädt, Hartmut Leuthold, Ian Grant Mackenzie, Tobin Dykstra, Eliot Hazeltine

**Affiliations:** 1 University of Tübingen, Germany; 2 University of Iowa, USA

**Keywords:** cognitive control, decision making, voluntary task switching, stimulus-response mapping, task representations

## Abstract

There has been an increasing interest in uncovering the mechanisms underpinning how people decide which task to perform at a given time. Many studies suggest that task representations are crucial in guiding such voluntary task selection behavior, which is primarily reflected in a bias to select task repetitions over task switches. However, it is not yet clear whether the task-specific motor effectors are also a crucial component of task representations when deciding to switch tasks. Across three experiments using different voluntary task switching (VTS) procedures, we show that a greater overlap in task representations with a task-to-finger mapping than task-to-hand mapping increases participants’ switching behavior (Exp. 1 and Exp. 2), but not when they were instructed to randomly select tasks (Exp. 3). Thus, task-specific stimulus-response associations can change the way people mentally represent tasks and influence switching behavior, suggesting that motor effectors should be considered as a component of task representations in biasing cognitive flexibility.

A central goal of cognitive psychology is to understand the underlying cognitive processes when performing a task. Beginning with Donders (1868/1969), researchers often investigate these processes by arbitrarily assigning responses to stimuli. For example, a simple choice task could require participants to make left or right key press responses with their left index or left middle finger depending on the color of a stimulus (e.g., blue color = left index finger response, green color = left middle finger response). Success in executing the task-specific arbitrary stimulus-response (S-R) links usually reflects an effective organization of the cognitive system by constraining the way in which perception is linked to action. In other words, the retrieval of an appropriate task-set allows participants to guide processing according to the S-R rules specified by the experimenter (e.g., Chen & Hsieh, 2015; Dreisbach & Haider, 2009; Goschke, 2000; Logan & Gordon, 2001; Sakai, 2008; Schumacher & Hazeltine, 2016).

Unfortunately, many everyday situations impose an additional challenge to our control systems because multiple task-sets are active as they are being performed in an overlapping or interleaved fashion. To address this issue in the laboratory environment, for example, participants could be instructed to perform a letter task (e.g., letter A = right index finger response, letter K = right middle finger response) in addition to the above-mentioned color task. Researchers have demonstrated that performance costs are typically observed when people switch tasks (e.g., performing the color task after the letter task) compared to when they repeat tasks (e.g., performing the color task again after just performing this task) (e.g., Jersild, 1927; for reviews, see Koch et al., 2018; Kiesel et al., 2010; Monsell, 2003; Vandierendonck et al., 2010).

Much progress has been made to illuminate the role of task representations when flexibly reconfiguring our cognitive system by externally signaling participants which of two tasks to perform in a given trial (e.g., a cue in advance of a trial indicates which task to perform, e.g., Logan & Schneider, 2006; Meiran et al., 2000). However, a full understanding of our cognitive system also requires us to consider the processes that are involved when voluntarily selecting tasks. With the present study, we aim to provide further insights into these processes by investigating how the effector-specific task mapping between stimuli and responses (S-R mappings) could potentially influence voluntary task switching behavior. This is an important endeavor on both theoretical and methodological grounds. Specifically, in light of the growing interest in investigating the cognitive mechanisms underlying voluntary behavior, many different studies infer the selected task in voluntary trials based on the task-specific effector chosen (e.g., Braem, 2017; Braun & Arrington, 2018; Fröber & Dreisbach, 2017; Frick et al., 2019; Irons & Leber, 2018; Reissland & Manzey, 2016; Mittelstädt et al., 2019; Wisniewski et al., 2016).

## Voluntary task switching

In voluntary task switching (VTS) experiments, participants are usually presented with a stimulus display in a given trial containing information associated with two independent task-sets. For example, participants are presented with a letter surrounded by a colored square and they can decide which of two tasks they want to perform in each trial (e.g., classifying the identity of the letter [letter task] or the color of the square [color task]). Critically, each task needs to be mapped to different response effectors so that the response can be used to infer which task was selected. Probably the most widely used task-to-effector mapping approach is to map each task to one hand (e.g., left index and middle finger = color task; right index and middle finger = letter task) (e.g., Arrington & Logan, 2004; 2005; Brüning et al., 2020; Chevalère et al., 2022; Frick et al., 2019; Jurczyk et al., 2019; Kessler et al., 2009; Mayr & Bell, 2006).

The most typical finding of VTS studies is a task repetition bias: Participants usually show a strong tendency to select the same task in trial *n* that they performed in trial *n-1* (e.g., Arrington & Logan, 2004; Jurczyk, et al., 2019; Kessler et al., 2009, Wong et al., 2022). Thus, in addition to the finding of switch costs in all VTS studies (i.e., slower reaction times and increased error rates when switching compared to repeating tasks), the difficulty of switching tasks is not only reflected in task performance but also in task selection behavior. The standard interpretation of the repetition bias relies on the idea that participants usually guide their task selection based on the most active task representation. More precisely, it is usually assumed that the previously applied task representation is usually the most active one in a given trial and hence participants are strongly biased to select task repetitions over task switches (e.g., Arrington & Logan, 2005; Dreisbach & Fröber, 2019; Mittelstädt et al., 2019; Vandierendonck et al., 2012). Critically, because participants tend to switch tasks rarely when left entirely free (e.g., Kessler et al., 2009; Arrington & Reiman, 2015; Wong et al., 2022), researchers have developed different experimental methods to increase switching behavior to provide a sufficient number of switches and repeats.

In the probably most widely used version of the VTS paradigm, participants are instructed to select the tasks in each trial randomly (e.g., Arrington & Logan, 2004; Brosowsky & Egner, 2021; Demanet et al., 2010; Mayr & Bell, 2006, Vermeylen et al., 2022). Although participants usually violate the instruction to select tasks randomly (i.e., they still select more often than chance task repetitions), they show reasonable switching behavior under these instructions (e.g., mean switch rates of >.30, cf. Arrington & Logan, 2004). Using this paradigm, a wealth of studies has provided important insights into the processes underlying volitional control (for a review, see Arrington, Reiman, & Weaver, 2014). Unfortunately, some of these findings also suggest that the randomness instruction may distort the cognitive processing that would have taken place without this instruction (e.g., Mittelstädt, Dignath et al., 2018; Liefooghe et al., 2010). For example, no asymmetric switch costs (i.e., higher switch costs when switching from a less practiced, weaker task to a well-practiced, stronger task) were observed when participants were instructed to select tasks randomly, whereas asymmetric switch costs were observed without these instructions (Liefooghe et al., 2010). This finding suggests that participants actively inhibit the previously selected task to (better) fulfill the instruction to randomly select a task (see also Lien & Ruthruff, 2008). Because less remaining task activation transfers from the previous to the current trial under the randomness instruction, switching behavior is also more frequent compared to when these instructions are not given (e.g., Arrington & Reiman, 2015; Liefooghe et al., 2010). In other words, the unbalanced (representational) activations of the two tasks (i.e., bias towards the potential switch task) are presumably less pronounced when participants are instructed to select a task randomly.

Other researchers refrain from using the randomness instruction, and instead change the structure of the task environment to increase switching behavior (e.g., Braun & Arrington, 2018; Dreisbach & Jurczyk, 2022; Fröber & Dreisbach, 2017; Jurczyk, et al., 2019; Qiao et al., 2022; Mittelstädt, Miller et al., 2018). For example, Fröber and Dreisbach (2017) have shown that reasonable switching behavior can be also observed when randomly intermixing voluntary (two task stimuli are presented) and forced task switching trials (only one task stimulus is presented) within blocks (e.g., mean switch rates of >.20 with 50% of forced task switching trials). As another example, Mittelstädt et al. (2019) have shown that a dynamic manipulation of repeat-versus-switch stimulus availability can also induce switching behavior without randomness instruction. Specifically, in their so-called self-organized task-switching paradigm, the onset of the task stimulus performed in trial *n-1* appeared delayed in trial *n* by a certain stimulus onset asynchrony (SOA), and this SOA increases with each additional repetition of the task. Thus, if participants wanted to repeat tasks, they had to wait longer for the repetition stimulus, whereas no waiting was required when they wanted to switch tasks. With this procedure, reasonable switching behavior was also achieved without the randomness instruction (e.g., mean switch rates of >.30 with an SOA step size of 50 ms, Mittelstädt, Miller et al., 2018; Mittelstädt et al. 2021).

The switching behavior in these two paradigms suggests that participants seem to adjust task activations based on (expected) changes of the task environment (e.g., Dreisbach & Fröber, 2019; Mittelstädt et al., 2019). Thus, these paradigms allow for counteracting the imbalance in task activations towards the potential switch task in a more natural manner—that is, without more or less directly instructing participants to switch tasks as is done with the randomness instruction.

## Effector-specific task representations in voluntary and externally controlled task switching

Regardless of which voluntary task-switching versions have been used, tasks were, in most cases, consistently mapped to different hands. However, a few studies have also mapped each task to homologous fingers (e.g., left index and right index finger = color task; left middle and right middle finger = letter task) (Chen & Hsieh, 2013; Serrien & O’Regan, 2019). The methodological choice of preferring a task-to-hand mapping over other effector mappings (e.g., a task-to-finger mapping) is usually not discussed by researchers. At first glance, the existing body of research does not suggest that the required task-specific effectors are a crucial component of task representations when deciding which task to select. First, the neural correlates of tasks seem to be represented within the prefrontal cortex (e.g., Sakai, 2008). If task representations guide task selections as suggested by previous VTS studies (e.g., Arrington, 2008; Dreisbach & Fröber, 2019; Mittelstädt et al., 2019), the decision of which task to perform should precede the act of selecting the upstream effector-specific motor action. Second, the repetition bias in a VTS study with randomness instruction was not observed when just hand switching instead of task switching was required (Vandierendonck et al., 2012). Thus, cognitive processes involved in switching between tasks (e.g., task-set reconfiguration) and not just effector-specific motor processes are apparently crucial in modulating switching behavior (Vandierendonck et al., 2012). Third and foremost, Chen and Hsieh (2015) conducted an electrophysiological VTS study with randomness instruction in which, among others, a task-to-hand mapping condition was compared with a task-to-finger mapping condition (with the task-to-effector mapping factor manipulated between participants). Their findings did not demonstrate that switch rates in the task-to-finger mapping condition (.46) differed significantly from the switch rates in the task-to-hand mapping condition (.43) (with *F*(1, 46) = .93, *p* = .34, cf. Chen & Hsieh, 2015).

Interestingly, however, a comparison of event-related related brain potentials (i.e., contingent negative variation) indicates that the task-to-effector manipulation affected how participants represented the two tasks in the study by Chen and Hsieh (2015). Specifically, only participants of the task-to-hand mapping condition showed an increased posterior switch-related negativity, suggesting that these participants engaged in advanced reconfiguration processes to update the (switch) task representation before stimulus onset. This might indicate that the spatial and anatomical task configuration in the task-to-hand mapping condition may help participants to create task representations that are more distinct, which in turn makes it easier for participants to adopt effortful preparatory strategies (for a similar suggestion, see Chen & Hsieh, 2015). However, even if so, the most crucial point for the present purpose is that their study does not suggest that such effector-specific task representations actually influence *voluntary switching behavior* (e.g., by switching tasks more often in the task-to-hand compared to task-to-finger condition).

Critically, the interpretation of null findings is, of course, problematic, and the requirement to follow randomness instructions in the study by Chen and Hsieh (2015) might have induced additional processes that might obscure (or even counteract) to observe potential effector-specific effects on voluntary task switching behavior. As mentioned above, Chen and Hsieh (2015) instructed participants to switch randomly in order to more directly compare potential switch-specific preparatory processes between the two mapping conditions. However, the instruction to randomly switch tasks implies *to not adapt* switching behavior to any experimental manipulation but to respond based on a mental representation of randomness. As mentioned above to better fulfill this randomness instruction, for example, inhibitory processes may more strongly suppress the activation of the previously applied task representation (e.g., Lien & Ruthruff, 2008). In other words, effector-specific task representations may only influence voluntary switching behavior when the recruitment of additional processes associated with the randomness instruction (e.g., inhibitory control) are reduced.

In line with this idea, various settings in which the task to be performed is externally controlled (e.g., a cue indicates which task to perform and hence no instruction to randomly select tasks) have shown an influence of effector-specific task representations on task performance (e.g., Cooper & Marí-Beffa, 2008; Hübner & Druey, 2008; Hsieh et al., 2014; Huestegge & Koch, 2013; Philipp & Koch, 2011). For example, Hsieh et al. (2014) have demonstrated that the time-consuming processes involved in a) switching between different response effectors (hand vs. foot responses) and in b) switching between different categories (shape vs. color) reflect at least partially shared mechanisms. Thus, the task-specific motor effectors can indeed play a role as a cognitive component of task representations to influence observable behavior (as measured in task performance) (e.g., Hsieh et al., 2014; Philipp & Koch, 2011). Of course, the cognitive components underlying task selection and task performance might at least partially differ (e.g., Arrington & Yates, 2009; Chen & Hsieh, 2013). However, the effector-specific effects on task performance can be taken as a further hint that effector-specific task representation may also affect voluntary *task selection* behavior when there is presumably less need for the recruitment of inhibitory control to fulfill the randomness instruction.

In sum, people usually guide their task selection behavior based on the most active task representation, as reflected in a preference to select task repetitions. Unfortunately, it is not yet clear whether the task-specific response effectors (e.g., task-to-hand vs. task-to-finger) must be considered as a component of the task representations to guide task selection behavior. Although behavioral measures from other contexts (e.g., task performance in instructed task-switching) suggest that the specific task-to-effector mapping affects our mental task representations, a previous study did not demonstrate that a task-to-effector manipulation influenced switching behavior (Chen & Hsieh, 2015). However, it seems premature to dismiss the possibility of effector-specific task representations guiding voluntary *task selection* behavior: Effector mappings may not have a strong impact on task representations (and hence switching behavior) when additional processes associated with the randomness instruction are at play (e.g., inhibitory control).

The major goal of the present study is to directly investigate whether effector-specific task mappings change the way we mentally represent tasks and influences voluntary task selection behavior using VTS paradigms without (Exp. 1: adaptive VTS paradigm; Exp. 2: hybrid free-forced VTS paradigm) and with randomness instruction (Exp. 3). Specifically, we investigate whether participants’ voluntary task switching behavior is influenced when tasks are mapped to different hands compared to when tasks are mapped to different fingers across the hands. Inspired by others (e.g., Chen & Hsieh, 2015), we reasoned that tasks can be more distinctly represented with a task-to-hand compared to task-to-finger mapping. Intuitively, a smaller overlap in task representations allows participants to keep the two tasks more equally activated because it reduces task confusion. Following others (e.g., Fröber & Dreisbach, 2017; Mittelstädt et al., 2021), this should counteract the strong preference to select task repetition and increase switching behavior. Thus, for our initial (preregistered) Experiment 1 using the adaptive VTS paradigm without randomness instruction, we assumed that less overlap in effector-specific task representation account would predict larger switch rates in the task-to-hand compared to the task-to-finger mapping condition. Alternatively, greater overlap in task representations with the task-to-finger than task-to-hand mapping may also increase switching behavior by increasing the availability of the corresponding switch task representation. Specifically, when the relevant task-set in trial n-1 is sufficiently activated to execute the selected task, overlap in task representations may also partially activate the irrelevant task-set in trial n-1—that is, the potential switch task-set in trial n. Finally, if the effector-specific manipulation does not influence switching behavior, it is apparently not necessary to consider response effectors as a cognitive component in guiding voluntary task switching behavior even when the randomness instruction is removed.

## Experiment 1

**Figure 1:**
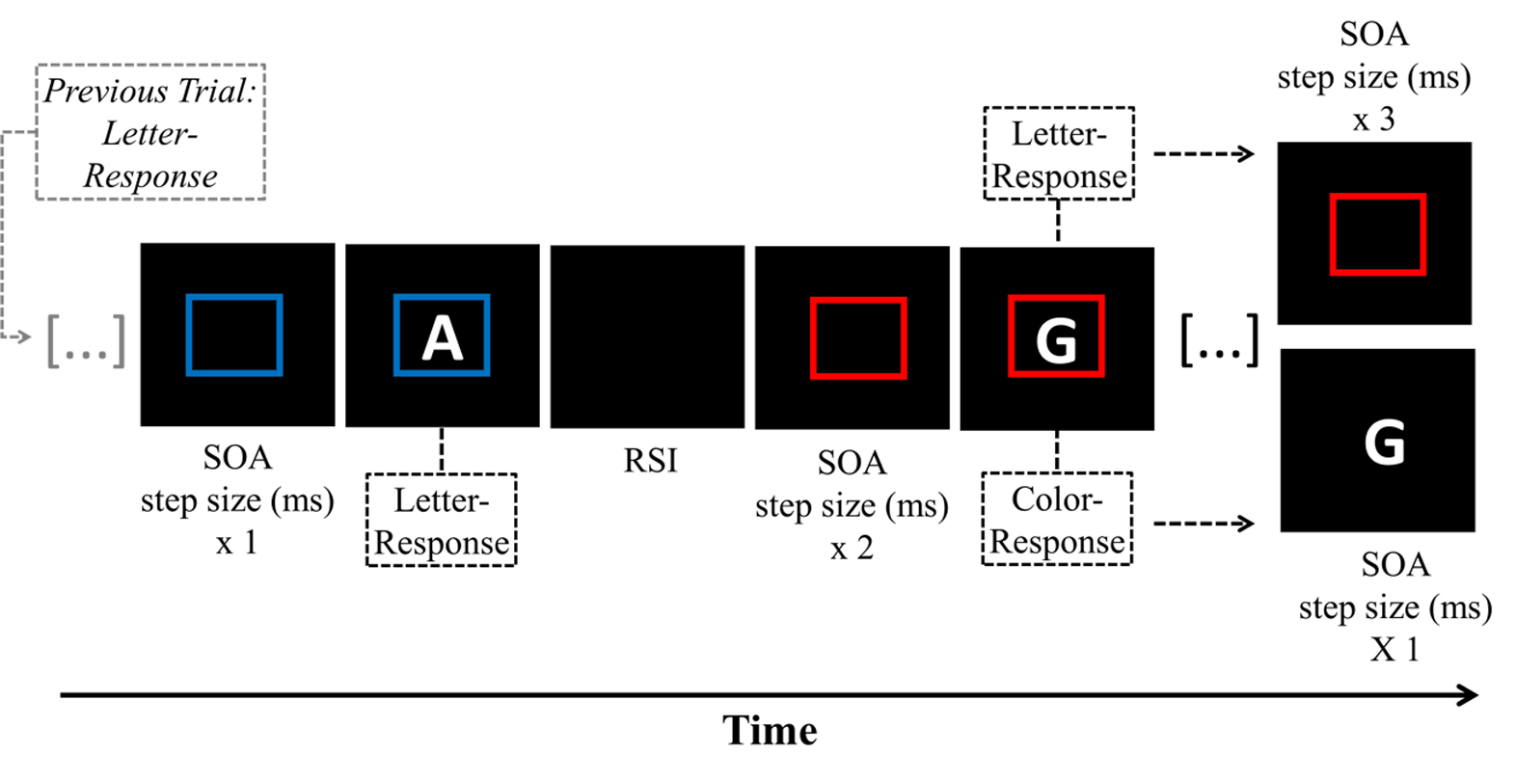
Typical trial sequences of the adaptive SOA blocks in Experiment 1. Stimuli were always centrally presented, but only the stimulus needed for a task switch was presented immediately following the response stimulus interval (RSI). The stimulus needed for a task repetition was presented with a stimulus onset asynchrony (SOA) step size (50 ms) that depended on the previous task selection history (i.e., how often this task was selected before).

In this experiment, we used the self-organized (voluntary) task-switching paradigm to investigate whether effector-specific task representation influences switching behavior in an environment without randomness instruction. Specifically, two stimuli (i.e., a letter and a colored square) associated with two independent task-sets were presented in a trial (i.e., color task and letter task). For half the blocks, tasks were assigned to the same hand (“task-to-hand”, e.g., performing the color task with the index and middle finger of the left hand), as is typical for VTS studies. In the other half of the blocks, tasks were assigned to the same fingers but different hands (“task-to-finger”, e.g., performing task 1 with the index fingers of the left and right hand). Throughout the experiment, participants could freely decide whether they want to perform the color task or the letter task by pressing a key with the corresponding task-dependent response effector. Thus, participants could decide whether they repeat or switch tasks in each trial and the response effector used in a given trial revealed whether a switch or repeat task decision was made. Following Mittelstädt et al. (2019), the stimulus onset for the task performed in trial *n-1* was delayed in trial *n* by an SOA of 50 ms. Thus, if participants wanted to repeat the previous task in trial n, they had to wait longer for the repetition stimulus. Moreover, the SOA increased further by 50 ms with each additional task repetition, whereas the stimulus needed for a task switch was always presented without delay (see Figure 1). Whenever a participant switched tasks, the SOA was reset to the first SOA step size, and a new sequence started.

### Method

#### Participants

32^1^ participants (27 female) were tested at the University of Tübingen, Germany. They ranged in age from 18 to 30 years (*M* = 20.9), and 25 were right-handed. All participants had normal or corrected-to normal vision, gave informed consent before testing and received course credit for participation.

#### Apparatus and stimuli

Stimulus presentation and data collection was controlled by E-Prime software. All stimuli were presented on the black background of the computer monitor. Stimuli were the four colors red (RGB[255,0,0]), blue (RGB[0,0,255]), green (RGB[0,255,0]), yellow (RGB[255,255,0]) for the color classification task. Stimuli were the four letters Q, G, K, L for the letter classification task. In each trial, a white letter (height: 6 mm, width: 5 mm) was centrally presented and surrounded by a colored square. Responses were key presses with the index and middle fingers of each hand on the numeric keyboard with the “1” (left index finger), “3” (right index finger), “4” (left middle finger) and “6” (right middle finger) keys. In task-to-finger blocks, one task was mapped to the left and right index fingers and the other task to the left and right middle fingers. In task-to-hand blocks, one task was mapped to the index and middle fingers of the left hand and the other task was mapped to the index and middle fingers of the right hand. The specific mappings (e.g., whether the color-task was mapped to the index or middle fingers in the task-to-finger blocks) was counterbalanced across participants.

#### Procedure

Each participant was tested in a single experimental session lasting approximately 45 min. Overall, each participant was tested in 14 blocks of 100 trials per block (1400 trials in total). Half of the participants were tested with the task-to-finger mapping for the first 7 blocks and with the task-to-hand mapping for the other 7 blocks, whereas the order was reversed for the other half of the participants. Different colors and letters were used in the two block conditions to avoid stimulus-specific learning confounds. Thus, in each task mapping condition, participants had to discriminate between two colors (= color task, e.g., task-to-finger-blocks: red vs. blue; task-to-hand-blocks: green vs. yellow) and two letters (= letter task, e.g., task-to-finger-blocks: Q vs. K; task-to-hand-blocks: G vs. L).

In each block, participants performed 50 trials in the color task and 50 trials in the letter task. For the color task, participants were required to classify the color of the square and for the letter task, they were required to classify the identity of the letter. They were instructed that they can decide in which order they want to perform these tasks, but they were also told that they should try to minimize the total time in each trial. After the practice blocks of each task-mapping condition (i.e., after block 1 and after block 8), the experimenter asked the participants if there were any questions and paraphrased the written instruction if needed. Specifically, participants received a German version of the following written instructions:

*“You have to respond to 50 colors and 50 letters in each block. Try to perform all of these 100 tasks as quickly and accurately as possible: Reaction time measurement starts with the onset of the first task (i.e., letter or colored square), and responses can be given after this onset. You can freely decide which task you want to perform in one trial, but try to respond as quickly and accurately as possible. If a #-sign or a grey square appear instead of one task, you have to wait to perform the other task.”*

The specific identity of the two task stimuli was randomly selected in each trial. After the necessary number of tasks of the same type was completed, placeholders were presented for the corresponding task (i.e., “#”-sign for the letter task or grey square for the color task) and key presses for this task were not recognized anymore. Thus, participants were then required to perform the remaining number of the other task until the block was completed.

Following the adaptive delay procedure by Mittelstädt et al. (2019), stimuli for the two tasks (letter surrounded by colored square) were only presented simultaneously in the first trial of a block, whereas in the remaining trials only the stimulus needed for a task switch was presented immediately. The other (potential repetition) stimulus was presented with an SOA that depended on the number of consecutive task repetitions. Specifically, the SOA linearly increased by an additional 50 ms with each task repetition until it was reset by a task switch (i.e., SOA step size = 50 ms).

Following correct responses, the stimulus display of the next trial was presented after a blank screen of 400 ms. Following incorrect responses, the response-stimulus interval (RSI) was set to 900 ms. During RSIs, participants received auditory feedback via headphones (i.e., low-pitched sounds for correct and high-pitched sounds for incorrect responses).^2^ Breaks between blocks were self-paced and participants received performance feedback after each block (i.e., mean trial time in milliseconds, calculated from the onset of the first stimulus until a response has been made, and number of errors). To ensure reasonable accuracy performance, participants were presented with an additional error screen in case of more than 10 errors within a given block. This screen indicated the correct stimulus-response mappings as well as that there were too many errors in this block for a fixed period of 60 s before participants could start the next block.

### Results

We first categorized the task performed on each trial based on the hand or finger used to respond. Then, trials were classified as repetition or switch trials on the basis of the task performed on trials *n* and *n–1*. Reported reaction times (RTs) always indicated the time from the onset of the stimulus related to the task that the participant performed until the key press.

The practice block of each mapping condition (i.e., block 1 and block 8)^3^ and the first trial of each block were excluded from any analyses. We then followed the same data trimming procedure as in previous studies (Mittelstädt et al., 2019). Thus, we then excluded any trials without the possibility of choosing between the two tasks (8.0%). Specifically, we excluded any remaining trials of a block when participants had already performed 50 trials for one task, because they were then “forced” to select and perform for the remaining trials the other task. Further, trials in which a response was given prior to stimulus onset (0.1%) and post-error trials (6.3%) were removed for all analyses. For RT and task selection analyses, 6.3% error trials were additionally excluded as well as trials with RTs less than 200 ms (0.4%) and greater than 3000^4^ ms (<0.1%). After our data trimming procedure, we examined the number of trials separately for each participant in each condition (i.e., switch vs. repetition trials separately for each mapping condition) in order to ensure that each participant had enough trials per condition to ensure a reasonable estimate of individual switch costs (> 10 trials per cell, cf. Mittelstädt et al, 2019). All participants had at least 15 valid trials in each of the specific condition.

#### Task selection: Switch rates

As can be seen from the first row in Table 1, mean switch rates were 4% larger in the task-to-finger (.33) compared to task-to-hand condition (.29) and a paired t-test indicated that this difference was significant, *t*(31) = 2.14, *p* = .041, *d_z_* = 0.38.

**Table 1:** Mean switch rates, mean reaction time (RT) and mean percentage errors (PE) as a function of trial transition (i.e., task switch vs. task repetition), mean RT switch costs and mean PE switch costs (i.e., task switch RT/PE–task repetition RT/PE) as a function of the two task mapping conditions and separately for each experiment (i.e., Experiment 1, 2, 3). Standard error of the means in parentheses.


MEASURE	EXPERIMENT 1		EXPERIMENT 2		EXPERIMENT 3	
		
	HANDS	FINGERS	HANDS	FINGERS	HANDS	FINGERS

Switch rate	.29 (.03)	.33 (.03)	.18 (0.01)	.24 (0.01)	.39 (.03)	.40 (.02)

Task switch RT	578 (15)	569 (15)	811 (23)	766 (20)	775 (19)	785 (20)

Task repetition RT	433 (7)	424 (9)	577 (15)	586 (17)	634 (19)	663 (19)

Switch cost RT	145 (13)	145 (17)	234 (17)	180 (12)	142 (15)	121 (16)

Task switch PE	6.8 (0.6)	7.2 (0.7)	7.0 (0.8)	7.6 (0.9)	4.0 (0.56)	4.9 (0.68)

Task repetition PE	5.5 (0.4)	6.5 (0.6)	2.7 (0.3)	3.0 (0.4)	3.3 (0.45)	3.2 (0.44)

Switch cost PE	1.3 (0.6)	0.7 (0.8)	4.2 (0.6)	4.6 (0.7)	0.8 (0.47)	1.8 (0.40)


##### Task performance: Reaction times (RT) and percentage errors (PE)

Following Mittelstädt et al. (2019), we calculated median switch costs for each participant.^5^ Mean median switch costs were numerically similar in the task-to-finger (145 ms) and task-to-hand (145 ms) mapping condition (see Table A1). An ANOVA on the mean median RTs with the within-subject factors of transition (repetition vs. switch) and mapping (task-to-finger vs. task-to-hand) revealed only a significant main effect of transition, *F*(1, 31) = 114.39, *p* < .001, *η_p_^2^* = .79 (with all other *p*s > .346 and *η_p_^2^*s < .03).

The last rows in Table 1 show that mean switch costs in PEs were descriptively slightly smaller in the task-to-hand (0.7%) than in the task-to-finger blocks (1.3%). An ANOVA parallel to the one conducted on median RTs revealed only (marginal) significant main effects of transition, *F*(1, 31) = 3.37, *p* = .076, *η_p_^2^* = .10, and mapping, *F*(1, 31) = 4.58, *p* = .040, *η_p_^2^* = .13, but no significant interaction (with *p* = .458 and *η_p_^2^* = .02). Mean PEs were larger in the task-to-finger (6.9%) compared to task-to-hand (6.1%) conditions.

### Discussion

We reasoned that task representations would overlap less in the task-to-hand compared to the task-to-finger mapping condition, leading to more frequent switching without randomness instruction. The results of this experiment do not demonstrate that switching tasks is easier (and consequently more common) when tasks are mapped to different hands than when tasks are mapped to different fingers. In fact, the results showed that switch rates were larger in the task-to-finger compared to task-to-hand condition—in the exact opposite direction of what we predicted. The additional analyses in Appendix B further demonstrate the stability of this finding by exploring the influence of mapping order and practice. Thus, while we continue to think that tasks were conceptualized as less distinct when they are mapped to fingers compared to hands, greater overlap in task representation facilitates engaging in switching behavior.

Unfortunately, some experimental particularities and other findings warrant some caution in interpreting the current results. Specifically, fine-grained analyses of switching behavior revealed that the average median switch SOA was only descriptively, but not reliably, smaller in the task-to-finger compared to task-to-hand mapping condition (see Appendix A). This suggests that the measure of switch SOA is less sensitive to detect potential effects of the task-to-effector manipulation—presumably because SOA varies only in discrete steps (i.e., 50, 100, 150 ms…), and this makes it difficult to detect effects when subjects switch in a less systematic manner (e.g., always switching after 3 switches). One might speculate that this might be due to additional random variations in task activation influencing switching behavior (i.e., independent of the effector-based manipulation or previous task choice). Another, not mutually exclusive, explanation is that the overlap in effector-specific task representations comes only into play to influence switching behavior when stimulus information associated with two tasks is presented. Note that with longer SOAs, task selection processes are probably already completed before participants are faced with two competing task stimuli. The finding of significantly larger switch rates in task-to-finger compared to task-to-hand blocks at the first SOA level seem in line with this speculation (see Appendix A). However, even if so, it seems fair to argue that the paradigm-specific particularity of varying SOA complicates the interpretation of the effector-specific differences in switch rates. Thus, in Experiment 2, we will replicate the finding with another VTS paradigm without randomness instruction.

The use of another VTS paradigm will also allow us to further explore another interesting finding of Experiment 1. Specifically, there were no differences in switch costs based on the task-to-effector manipulation. As briefly mentioned in the introduction, a number of studies using settings in which task(s) to perform were externally specified by the environment/experimenter have actually found effector-specific effects on task performance. To explain the apparent discrepancy of effector-based effects on task selection and task performance in the present setting, it seems necessary to postulate that the processes underlying task performance and task selection differ in voluntary task switching settings (e.g., Arrington & Yates, 2009). To shed further light on this issue, we will explore task performance in the following Experiment 2 to see whether the absence of effector-specific effects on task performance generalizes to another VTS paradigm.

## Experiment 2

The previous results suggest that overlap in effector-specific task representation leads to increased switching behavior. However, as discussed above, this interpretation was post-hoc and experimental particularities of the adaptive VTS paradigm might have somehow encouraged participants to select tasks in a way that is atypical in other VTS paradigms. The purpose of Experiment 2 was to check whether larger switch rates with task-to-finger compared to task-to-hand mapping would also be obtained when using another VTS paradigm without randomness instruction; specifically, the hybrid paradigm in which voluntary (“free”) and non-voluntary (“forced”) trials are combined (e.g., Chiu et al., 2020; Jurczyk et al., 2021; Mendl & Dreisbach, 2022). Thus, in Experiment 2, tasks will again be mapped to hands versus fingers, but in each block, forced trials (i.e., only one stimulus is presented) and free trials (i.e., two stimuli are presented) are randomly intermixed (free-forced ratio of 50:50). Our focus concerns the free task choice trials. We predict that switch rates will be larger in the task-to-finger compared to task-to-hand mapping condition. Statistically, this should result in a significant effect (*p* < .05) when conducting a paired *t*-test between the two mapping conditions on the proportion of switches. Beyond a confirmatory test of effector-specific modulation of switch rates, we will also explore and report the possible impact on task performance in the main text and complementary (paradigm-specific) analyses in Appendix C.

### Method

#### Participants

As preregistered, 80 participants^6^ were recruited (via Prolific) and tested online in a single session using jsPsych (de Leeuw, 2015). The data of 10 participants were excluded due to fewer than 10 valid trials in at least one of the eight experimental conditions for the task performance analyses of free choice trials (i.e., switch/repetition X finger/hand mapping).^7^ The remaining 70 participants (28 female) ranged in age from 18 to 47 years (*M* = 28.33), and 66 were right-handed.

#### Apparatus and stimuli

The experiment was conducted online using the JavaScript library jsPsych (de Leeuw, 2015). The stimuli were the same as in Experiment 1, but there was a difference in response keys. Specifically, in this experiment, hands were placed on the keyboard in a more standard way as is usually done in studies using VTS paradigms. In Experiment 1, hands were placed on a numeric keyboard. Although this was done so that each individual response effector had the same distance from each other, it could have encouraged participants to use additional task selection strategies than they normally do with a standard hand-on keyboard configuration. Consequently, responses were key presses with the index and middle fingers of each hand with the “Q” (left middle finger), “W” (left index finger), “O” (right index finger), and “P” (right middle finger) keys on standard QWERTZ or QWERTY keyboards.

#### Procedure

Except as described, the procedure was the same as in Experiment 1. Participants were tested in 14 block of 84^8^ trials per block with blocks of the specific task-to-effector mapping counterbalanced across participants (e.g., half of the participants will be tested with the task-to-finger mapping for the first 7 blocks and with the task-to-hand mapping for the last 7 blocks). Following the standard procedure of the hybrid free-forced paradigm (e.g., Fröber & Dreisbach, 2017; Jurczyk et al., 2021), in 42 trials of each block only one task stimulus (i.e., 21 trials only a letter and in 21 trials only a colored square) was presented whereas in the remaining 42 trials of each block both task stimuli were presented (i.e., 42 free-choice-trials and 42 forced-choice-trials). Participants were instructed that if only one stimulus was presented, they are required to perform the corresponding task. However, if two stimuli are presented, they were told that they can decide which task of the two tasks they want to perform. Specifically, participants received a German or English version of the following written instructions:

*“When only on task stimulus is presented (i.e., letter or colored square) you have to perform the corresponding task. However, if both task stimuli are presented you can freely decide which task you want to perform in this trial.”*

Thus, in contrast to the previous experiment, in free choice trials stimuli for the two tasks were always presented simultaneously.

### Results

As preregistered, we followed the same data preparation procedure as in Experiment 1. The first two blocks of each task-mapping condition were considered practice (i.e., block 1 & 2 and block 8 & 9) and excluded from any data analyses. Further, post-error trials were removed for all analyses (4.0%). For RT and task selection analyses, error trials were additionally excluded (4.0%) as well as trials with RTs less than 200 ms and greater than 2000 ms (1.4%).

#### Task selection (confirmatory analyses): Switch rates

In line with our central hypothesis, switch rates were significantly larger in the task-to-finger (.24) compared to task-to-hand mapping (.18) condition (see Table 1), *t*(69) = 7.22, *p* < .001, *d_z_* = 0.83.

#### Task performance in free-choice trials (exploratory analyses): Reaction times (RT) and percentage errors (PE)

Table 1 shows switch costs in free choice trials separately for each mapping condition. The RT-ANOVA with the within-subject factors of transition (repetition vs. switch) and mapping (task-to-finger vs. task-to-hand) revealed a significant main effect of transition (i.e., overall switch costs of 207 ms), *F*(1, 69) = 256.72, *p* < .001, *η_p_^2^* = .79. Furthermore, there was also a marginal significant effect of mapping, *F*(1, 69) = 3.65, *p* = .060, *η_p_^2^* = .05, reflecting slightly smaller RTs in the task-to-finger compared to task-to-hand condition (676 ms vs. 694 ms). Interestingly, there was also a significant interaction, *F*(1, 69) = 15.39, *p* < .001, *η_p_^2^* = .18. As can be seen in Table 1, switch costs were larger in the task-to-hand compared to task-to-finger condition. The PE-ANOVA only yielded a significant main effect of transition (i.e., overall switch costs of 4.41%), *F*(1, 69) = 73.96, *p* < .001, *η_p_^2^* = .52 (with all other *p*s > .342, *η_p_^2^* < .02).

### Discussion

In line with Experiment 1, switch rates were again larger in the task-to-finger compared to the task-to-hand condition. Thus, the results provide further support for the idea that a larger overlap in effector-specific task representations with task-to-finger compared to task-to-hand mapping increases switching behavior in a VTS paradigms without randomness instructions (here, the hybrid free-forced paradigm, Fröber & Dreisbach, 2017). Interestingly, in contrast to Experiment 1, there were also effector-specific effects on task performance – that is, RT switch costs were smaller in the task-to-finger compared to task-to-hand mapping conditions. As can be seen in Appendix C, a similar effect was also found in task performance of forced choice trials for which there was the same number of switch and repetition trials in each mapping condition. Together, the task performance results suggest that the free-choice effect is not simply a by-product of practice differences due to biased task choices. Against the background of the task choice results, it seems that there may also be partially similar processes underlying task performance and task choice behavior, at least, in the hybrid free-forced task switching environment.

## Experiment 3

In the previous two experiments, we investigated whether effector-specific task representations influence switching behavior in task environments without randomness instruction. In Experiment 3, we will test whether increased switch rates with task-to-finger compared to task-to-hand mapping could also be obtained even when using the classic VTS paradigm with randomness instruction (e.g., Arrington & Logan, 2004). A study by Chen and Hsieh (2015) did not observe significantly larger switch rates between participants using a task-to-finger compared to a task-to-hand mapping. As mentioned in the introduction, we reasoned that additional processes associated with the randomness instruction may at least partially distort the effector-specific representational activity normally seen without these instructions to influence switching behavior (e.g., inhibitory processes). However, it should also be emphasized that the electrophysiological study by Chen and Hsieh (2015) was not designed to investigate switch rate differences between the task-to-effector conditions (between-subject factor) so that the non-significant difference might be due to a lack of power. Furthermore, some particularities in their design may also influence potential effector-specific switching behavior (e.g., use of a bivalent stimulus in their study vs. use of univalent stimuli in our study). In any case, it seems useful to directly test whether a significant difference in switch rates between the two mapping conditions will be observed when the same tasks as in the previous experiments are used in a standard VTS setting. Beyond a confirmatory test of effector-specific modulation of switch rates in Experiment 3, we also further explored and report whether the size of a potential modulation is different from the one in Experiment 2 (without randomness instruction, see Appendix D). Of course, we will again explore and report the possible impact on task performance.

### Method

#### Participants

As preregistered, 80 participants were recruited (via Prolific) and tested online. The data of 28 participants were excluded due to fewer than 10 valid trials after the data trimming procedure in at least one of the experimental conditions for the task performance analyses.^9^ The remaining 52 participants (28 female) ranged in age from 19 to 44 years (M = 27.62), and 51 were right-handed.

#### Apparatus and stimuli

The apparatus and stimuli were the same as for Experiment 2.

#### Procedure

The procedure was the same in Experiment 2 except for the following changes. First, two stimuli were presented in each trial. Thus, there were only free choice trials and participants were told that they can decide which of the two tasks they want to perform, but they should select both tasks equally often and in a random sequence. Specifically, participants received a German version of the following written instructions (cf. Arrington & Logan, 2004):

*“You should perform each task (i.e., letter or color) on about half of the trials and you should perform the task in a random order. For example, imagine that you have a coin that said “color-task” on one side and “letter-task” on the other. Try to perform the task as if flipping the coin decided which task to perform. So sometimes you will be repeating the same task and sometimes you will be switching tasks. We don’t want you to count the number of times you’ve done each task or alternate strictly between tasks to be sure you do each one half the time. Just try to do them randomly.”*

### Results

As preregistered, we followed the same data preparation procedure as in the previous two experiments. The first two blocks of each task-mapping condition were considered practice (i.e., block 1 & 2 and block 8 & 9) and excluded from any data analyses. Further, post-error trials were removed for all analyses (3.9%). For RT and task selection analyses, error trials were additionally excluded (3.9%) as well as trials with RTs less than 200 ms and greater than 2000 ms (2.3%).

#### Task selection (confirmatory analyses): Switch rates

Switch rates were descriptively only slightly larger in the task-to-finger (.40) compared to task-to-hand mapping (.39) condition (see Table 1) and a paired *t*-test indicated no significant difference, *t*(51) = 0.33, *p* = .743, *d_z_* = 0.05.

#### Task performance in free-choice trials (exploratory analyses): Reaction times (RT) and percentage errors (PE)

The RT-ANOVA with the within-subject factors of transition (repetition vs. switch) and mapping (task-to-finger vs. task-to-hand) only revealed a significant main effect of transition (i.e., overall switch costs of 131 ms), *F*(1, 51) = 99.71, *p* < .001, *η_p_^2^* = .66 (with all other *p*s > .126, *η_p_^2^* < .06). The PE-ANOVA only yielded a significant main effect of transition (i.e., overall switch costs of 1.26%), *F*(1, 51) = 19.70, *p* < .001, *η_p_^2^* = .28 (with all other *p*s > .129, *η_p_^2^* < .05).

### Discussion

In contrast to the previous two experiments, there was no evidence that the task-specific response effector influences switching behavior in the VTS paradigm with randomness instruction. Notably, this finding is in line with a previous VTS study in which participants were instructed to randomly select tasks (Chen & Hsieh, 2015). As mentioned in our introduction, we suggest that the discrepancy between these between-experiment patterns^10^ may reflect additional processes associated with the randomness instruction. In other words, participants may differentially represent tasks as a function of the effector mapping condition in Experiment 3. However, following the randomness instruction counteracts potential effects on switching behavior which would normally emerge without this instruction as in the previous two experiments.

## General Discussion

In the present study, we tested the hypothesis that the task-specific motor effector can change the way mental task representations influence voluntary task selection behavior. For this purpose, the responses for the same task were either mapped to same hand or same fingers. We reasoned that the task can be more distinctly represented with a task-to-hand compared to task-to-finger mapping. In two experiments using different VTS procedures without randomness instruction, we observed larger switching behavior with task-to-finger compared to task-to-hand mapping. This suggest that the greater overlap in task representations with the task-to-finger mapping increases switching behavior. Thus, task-specific motor effectors should be considered as a component of task representations to bias cognitive flexibility.

The finding that rather late processing components (i.e., motor effectors) can influence voluntary task choice behavior extends previous studies suggesting rather early (e.g., perceptual) task choice biases (e.g., Mittelstädt et al., 2022; Arrington, 2008; Mayr & Bell, 2006). For example, some studies have shown that switching behavior increases for stimuli switches than repetitions, suggesting that task-specific perceptual features can strengthen task representations to guide task choices (e.g., Demanet et al., 2010; Mayr & Bell, 2006). Here, we show that it is critical how task-specific information is linked to the upstream effector-specific motor action since this can already bias voluntary task choice behavior–presumably due to changes in mental task representation as has been suggested in the context of externally controlled task processing (e.g., Hazeltine & Schumacher, 2016; Philipp & Koch, 2011).

While we hope that this finding helps the further development of accounts emphasizing the modulation of task representations along the stability-flexibility continuum (e.g., Brosowsky & Egner, 2021; Dreisbach & Fröber, 2019), there is also one methodological implication. Participants typically show a strong avoidance of selecting task switches and this avoidance is sometimes even robust to environmental changes that make switching more attractive (e.g., Mittelstädt et al., 2019). Thus, VTS studies that require that participants to engage in sufficient switching behavior without randomness instructions may also consider mapping the tasks to different fingers instead of hands as has been done in the majority of previous VTS studies. In this regard, it is also important to emphasize that some caution needs to be applied when interpreting influences on task choice behavior in the VTS paradigm with randomness instruction. While findings obtained in this paradigm can certainly generalize to paradigms without randomness instruction (e.g., Mittelstädt et al., 2022), additional processes to follow the requirement to select the task randomly might also make it difficult to observe any effects of task choice behavior. Moreover, the relatively large dropout of participants who did not engage in switching despite the randomness instruction in the present web-based experiment (i.e., 28 of 80 participants) suggests that it is also more challenging in an online- than lab-based setting to ensure that participants have actually read and understood the instructions. Thus, researchers should rather opt for VTS paradigms which induce switching behavior with contextual environmental changes (e.g., Jurczyk et al., 2022; Mendl & Dreisbach, 2022).

## Data Accessibility Statement

Raw data of all experiments and stage 1 protocol are available via the Open Science https://osf.io/aeq6y/.

## Additional File

The additional file for this article can be found as follows:

10.5334/joc.255.s1 Appendixes. Appendixes A to D.

## Footnotes

As can be seen in our preregistration (https://osf.io/j6ya7), we had initially planned to test 48 participants. However, we had decided to stop data collection due to Covid-19 restrictions. Note that the sample size of 48 participants was somehow arbitrary set, because we had no information about the possible effects size and we wanted to compensate for potential dropouts. Note, however, that no participants had to be excluded when applying the same data exclusion criteria as in a previous study (Mittelstädt et al., 2019; Mittelstädt et al., 2021).

In the first practice block, participants were additionally presented with the correct stimulus-responses mapping after an erroneous trial for a self-paced time.

Note that mean switch rates were noticeably lower for both block 1 (.19) and block 2 (.18) than for the other twelve blocks (range: .29 to .36) suggesting that participants also needed block 2 to adapt their behavior to the structure of the task environment. Although we will consider this issue in our registered Experiments 2 and 3 by additionally excluding block 2 and block 9 for our data analyses, we decided to keep these blocks for the analyses in Experiment 1 in order to have an equal number of blocks for our exploration of mapping order and practice described in Appendix B. However, we would like to note that additionally excluding block 2 and 9 revealed very similar results, including a significant difference in switch rates between the two mapping conditions, *t*(31) = 2.27, *p* = .030, *dz* = 0.40.

Based on visual inspection of the RT frequency distribution, a cut-off of 2000 ms seem more appropriate. However, we decided to stick with our preregistered analyses, but we will use a cut-off of 2000 ms for the next two planned experiments. Note also that very similar results were also found when using a cut-off of 2000 ms for Experiment 1.

Note that there was also no significant interaction when using means instead of medians (with mean switch costs of 198 ms and 187 ms for the hand and finger condition, respectively).

A power analysis to detect an effect of similar size as the one found in switch frequencies in the task-to-finger compared to task-to-hand response mapping condition in Experiment 1 with a power level of 80% (90%) and a significance level of 5% (one-sided) would have suggested 45 (61) participants. Furthermore, as was mentioned in our preregistration, we decided to increase the lab-based planned sample size from 64 to 80 participants to compensate for an increase in effect variability in online compared to laboratory settings of about 5% (Miller et al., 2018).

As preregistered, we excluded participants with no or few trials (i.e., <10) in some conditions to obtain reasonable estimates for the variables in our task performance analyses. Because the result pattern was very clear in Exp. 2 and Exp. 3 and for practical reasons, we decided to not replace the data of these participants, contrary to what we had stated in our preregistered report. We would also like to note that in all experiments, the critical results for task choice behavior were very similar when including all participants or applying a less strict criterion (i.e., only excluding participants with less than 5 valid trials in some conditions).

Please note that we had decided to reduce the number of trials per block from 100 to 84 trials after some pilot testing to achieve a feasible overall duration of the *online* experiment.

Note that the results were very similar when excluding two additional participants with mean switch rates lower than 10% (i.e., participants who clearly violated the instruction to select tasks randomly).

Note that an additional comparison of Experiment 2 and Experiment 3 revealed a significant modulation of the mapping effect as a function of experiment (see Appendix D), indicating that there were indeed experiments-specific effects of the mapping manipulation.
