# Supplementary material for: The Role of Effector-Specific Task Representations in Voluntary Task Switching
Source: J Cogn. 2023 Jan 13;6(1):9. doi: 10.5334/joc.255 (PMC9838227; doi:10.5334/joc.255)
Supplement: Appendixes. — Appendixes A to D. [file joc-6-1-255-s1.pdf]

Appendix A (Experiment 1)

In this appendix, we describe additional analyses beyond our central comparison of switch frequencies between the two mapping conditions. Specifically, we also compared *switch SOAs* as another dependent variable to capture voluntary switching behavior. Switch SOAs describe how much extra switch stimulus availability is needed to elicit task switches in individual runs and can be calculated by examining the SOAs in trials in which participants decided to switch. The possibility of measuring switching behavior on a time scale allowed additional insights into how participants adapt to the task mapping condition. In general, we expected that potential *higher switch rates* in task-to-hand compared to task-to-finger blocks should also be reflected in switch SOAs (i.e., *lower switch SOA* in task-to-hand than task-to-finger). Moreover, the measure of switch SOA also allowed us to explore how switching limitations (as measured by switch costs) were related to switching behavior by comparing these two measures. In previous studies, we observed that participants tended to switch tasks when the availability benefits matched switch costs (i.e., size of switch costs = switch of SOA), and we wanted to know whether this finding can also be observed in the present modified task environment (e.g., different tasks) and across different conditions (i.e., task-to-hand and task-to-finger condition).

Results

Task selection: Switch SOAs. To examine in more detail switching behavior as a function of SOA, we plotted the corresponding cumulative distribution function (CDF) of switch SOA averaged over participants separately for each mapping condition. Thus, these CDFs describe the distribution of switches out of the trials in which switches did occur (i.e., the number of repetition trials plays no role). As can be seen in Figure A1, there was already a large difference in switch rates between the task-to-hand mapping condition (.13) and task-to-finger mapping condition (.19) at the first SOA level and this difference was significant, $t(31) = 2.89$, $p = .008$, $d_z = 0.51$.

We then computed each participant's individual (interpolated) median switch SOA as a summary measure of task selection behavior as a function of SOA (cf., Mittelstädt et al., 2019). Note that the CDFs in Figure A1 also illustrate the corresponding average median switch SOAs (i.e., points when the CDFs cross the .50 values). Although participants switched in average at a smaller SOA in the task-to-finger (150 ms) compared to task-to-hand (164 ms) condition, this difference was not significant, $p = .267$, $d_z = 0.20$.

Relation between task selection and task performance. Finally, we also compared the size of switch costs with switch SOA separately for each mapping condition. Paired t-tests indicated that the difference between these measures were neither significant in the task-to-hand condition (with $p = .340$ and $d_z = 0.17$) nor in the task-to-finger condition (with $p = .811$ and $d_z = 0.04$). Switch cost-SOA matches has been also observed in previous studies using this paradigm (Mittelstädt, Miller et al., 2018; Mittelstädt et al., 2019). Thus, these analyses extend our recent suggestion that participants approximately switched tasks when the size of switch SOAs matched their switch costs in both task-to-finger and task-to-hand blocks. For completeness, we also investigated whether the chosen cost-SOA trade-off differed between the mappings condition. However, an ANOVA with the factors of measure (i.e., SOA vs. RT) and mapping revealed no significant interaction ($p = .486$; $\eta_p^2 = .02$).

Finally, we also plotted the individual median switch costs against the individual mean switch rates (see Figure A2) as well as against the individual median switch SOAs (see Figure 2B) separately for the two task-to-effector mapping conditions. For switch costs against switch rates, there were significant negative correlations in both task-to-hand, $r(30) = -.39$, $p = .026$ and task-to-finger blocks, $r(30) = -.59$, $p < .001$. For switch costs against switch SOA,

there was a non-significant positive correlation in task-to-hand blocks, $r(30) = .17$, $p = .359$ and a significant positive correlation in task-to-finger blocks, $r(30) = .40$, $p = .025$.[1]

[1] Note that we also checked the correlational findings when excluding the one participant with negative switch costs and strong switching behavior: For switch costs against switch rates, there was a negative but not reliable correlation in task-to-hand, $r(29) = -.28$, $p = .128$ and a significant negative correlation in task-to-finger blocks, $r(29) = -.43$, $p = .015$. For switch costs against switch SOA, there was a non-significant and only small positive correlation in task-to-hand blocks, $r(29) = .10$, $p = .603$ and a marginal significant positive correlation in task-to-finger blocks, $r(29) = .34$, $p = .058$. Note also that mean switch rates in the task-to-finger blocks (.29) were still larger than the ones in the task-to-hand blocks (.27) when excluding this participant, albeit, this difference was only marginally significant, $t(30) = 1.96$, $p = .060$.

Appendix B (Experiment 1)

In this appendix, we present complementary analyses to explore whether and how other factors potentially modulated any effects of our central task mapping manipulation. Specifically, we investigated the influence of mapping order (i.e., task-to-hand blocks followed by task-to-finger blocks [HF-Order] vs. task-to-finger blocks followed by task-to-hand blocks [FH-Order]) and practice.

Task mapping order

The top part of Table B1 shows the corresponding measures as a function of task mapping condition separately for participants with HF- and FH-mapping order.

Task selection: Switch rates. As can be seen from Table B1, mean switch rates were considerably larger for the finger compared to the hand condition for HF-Order participants. In contrast, mean switch rates were slightly larger for the hand compared to the finger condition. An ANOVA including the within-subject factor task mapping and the between-subject factor mapping order revealed a significant interaction, $F(1, 30) = 17.03$, $p < .001$, $\eta_p^2 = .36$, in addition to a significant main effect of task mapping, $F(1, 30) = 6.92$, $p = .013$, $\eta_p^2 = .19$. Follow-up comparison revealed a significant difference between mapping conditions for HF-order participants ($p = .001$, $d_z = 1.10$), but no significant difference between these conditions for FH-order participants ($p = .263$, $d_z = 0.29$).

Task selection: Switch SOAs. The mean median switch SOAs displayed for the corresponding conditions in Table B1 resembled the pattern found for switch rates: Participants with HF order switched in average at a smaller SOA in the task-to-finger compared to task-to-hand condition, whereas participants with FH order switched at a slightly smaller SOA in the task-to-hand compared to task-to-finger condition. A mixed ANOVA parallel to the one conducted on mean switch rates only revealed a significant interaction, $F(1, 30) = 4.60$, $p = .040$, $\eta_p^2 = .13$. Follow-up comparison revealed a marginal significant difference between mapping conditions for HF-order participants, $t(15) = 1.87$, $p = .081$, $d_z =$

0.34, and no significant difference between these conditions for FH-order participants (p = .312, d_z = 0.18).

Task performance: Reaction times (RT). A mixed ANOVA with the factors transition, task mapping condition, and mapping order revealed, in addition to a significant main effect of transition, $F(1, 30) = 110.70$, $p < .001$, $\eta_p^2 = .79$, a significant three-way interaction, $F(1, 30) = 5.06$, $p = .032$, $\eta_p^2 = .14$. As can be seen from Table B1, this interaction reflected the finding that switch costs were descriptively larger for task-to-hand compared task-to-finger blocks for HF-order participants whereas this pattern was reversed for FH participants. Follow-up ANOVAs separately for each order group of participants revealed that the mapping x transition interaction was neither significant for HF-order participants (p = .136, $\eta_p^2 = .14$) nor for FH-order participants ($p = .129$, $\eta_p^2 = .15$).

Task performance: Percentage errors (PE). The mean PE pattern resembled the one found for mean median RT. In addition to a significant main effect of task mapping, $F(1, 30) = 4.49$, $p = .043$, $\eta_p^2 = .13$), there was also a marginal significant three-way interaction for the corresponding 3-way mixed ANOVA on PEs, $F(1, 30) = 4.17$, $p = .050$, $\eta_p^2 = .12$). Follow-up ANOVAs separately for each order group of participants revealed a marginal significant mapping x transition interaction for HF-order participants, $F(1, 15) = 4.45$, $p = .052$, $\eta_p^2 = .23$, whereas this interaction was not significant for FH-order participants ($p = .411$, $\eta_p^2 = .05$).

Relation between task selection and task performance. Finally, we compared the size of switch costs RT with switch SOA separately for each mapping condition for participants with HF-order and FH-order. The corresponding paired t-test revealed no significant effects (all ps > .478 and all $d_z < 0.17$). ANOVAs with the factors of measure (i.e., SOA vs. RT) and mapping (task-to-hand vs. task-to-finger) separately for each mapping condition revealed no significant interaction for participants with HF-order ($p = .793$; η_p^2 = .01) and FH-order ($p = .291$; $\eta_p^2 = .07$).

Summary. The analyses involving the between-subject factor mapping order revealed that mapping order did indeed modulate the effects of the task-mapping manipulation on switching behaviour. More precisely, there was a significant increase of 10% in switch rates for task-to-finger compared to the task-to-hand mapping blocks for the group of participants with HF-order, whereas for the group of participants with FH-order, there was a non-significant decrease of 2% in switch rates for task-to-finger compared to the task-to-hand mapping blocks. It should be emphasized, however, that this finding only demonstrates the presence of a general increase of switching behavior over the course of the experiment. Specifically, for the HF-group, this general switch rate increase additionally boosts the switch rate increase when the task mapping changes from hands to fingers. For the FH group, this general switch rate increase counteracts the switch rate decrease that happens when the task mapping changes from fingers to hands.

Practice order

The previous analyses indicate that switching behavior changes over the course of the experiment. We then wondered whether the effect of task-mapping on switch rates (and also the other findings) was already present in the first blocks of the task-specific mapping condition or whether participants needed some time to practice and explore switching behavior with the instructed task mapping until they actually differently adapted their switching behavior to the two different mappings. To investigate this issue, we separated each task mapping condition into an early (first) part and a late (second) part. Specifically, from the total of 12 experimental blocks (with one mapping condition from blocks 1-6 and the other mapping condition from blocks 7-12), blocks 1-3 and blocks 7-9 were classified as the first part, whereas blocks 4-6 and blocks 10-12 were classified as the second part. We then reanalyzed all results including the within-subject factor of part.

Task selection: Switch rates. As can be seen from Table B1, mean switch rates were larger in the task-to-finger compared to the task-to-hand condition in both the first and second

part of the blocks. An ANOVA including the within-subject factors task mapping and part (first, second) revealed in addition to a significant main effect of task mapping, $F(1, 31) = 4.40$, $p = .044$, $\eta_p^2 = .12$, a significant main effect of part, $F(1, 31) = 15.03$, $p = .001$, $\eta_p^2 = .33$: Mean switch rates were larger for task-to-finger compared to task-to-hand blocks (.29 vs. .33) and mean switch rates were larger for the second compared to first part of block (.33 vs. .29). The interaction was not significant ($p = .761$, $\eta_p^2 < .01$) but for completeness we also conducted and report the follow-up comparison between mapping conditions separately for the first and second part: The mapping difference was only significant in the second part, $t(31) = 2.20$, $p = .035$, $d_z = 0.39$, but not in the first part of blocks ($p = .110$, $d_z = 0.20$).

Task selection: Switch SOAs. Not surprisingly, the mean median switch SOAs displayed for the corresponding conditions in Table B1 resembled the pattern found for switch rates: For both the first and second part of blocks, switch SOAs were larger for the task-to-hand compared to the task-to-finger condition. An ANOVA parallel to the one conducted on mean switch rates revealed no significant effects (all *p*s > .129 and all $\eta_p^2 < .08$). Follow-up paired t-tests revealed also no significant differences in switch SOAs between the mapping conditions neither for the first ($p = .234$, $d_z = 0.21$) nor for the second part ($p = .352$, $d_z = 0.16$).

Task performance: Reaction times (RT). An ANOVA with the factors transition, task mapping condition and block part revealed in addition to significant main effect of transition, $F(1, 31) = 60.37$, $p < .001$, $\eta_p^2 = .66$) and part, $F(1, 31) = 5.03$, $p = .032$, $\eta_p^2 = .14$, only a significant two-way interaction transition x part, $F(1, 31) = 19.69$, $p < .001$, $\eta_p^2 = .39$. This interaction indicated that switch costs were larger for the first (182 ms) than for the second part (100 ms) of blocks. Follow-up ANOVAs separately for each block part revealed no significant mapping x transition interaction for the first ($p = .683$, $\eta_p^2 < 01.$) or second part of blocks ($p = .282$, $\eta_p^2 = .04$).

Task performance: Percentage errors (PE). The mean PE pattern resembled the one found for mean median RT. The corresponding 3-way ANOVA revealed only significant main effects of transition, $F(1, 31) = 4.31$, $p = .046$, $\eta_p^2 = .12$, and task mapping, $F(1, 31) = 5.26$, $p = .029$, $\eta_p^2 = .14$. As was already reported in the main analyses, the main effect of task mapping indicated slightly larger PEs for task-to-finger than task-to-hand blocks. Follow-up ANOVAs separately for block part revealed significant main effects of transition, $F(1, 31) = 5.56$, $p = .025$, $\eta_p^2 = .15$, and task mapping, $F(1, 31) = 4.73$, $p = .037$, $\eta_p^2 = .13$, for the first part of blocks, but no significant effects for the second part of blocks (all *p*s > .162 and all $\eta_p^2 < .07$).

Relation between task selection and task performance. Finally, we compared the size of switch cost RT with switch SOA separately for each mapping condition for the first and second part of the blocks. Except for a marginal significant difference between switch costs and switch SOAs for the second part of the task-to-hand mapping condition, $t(31) = 1.88$, $p = .070$, $d_z = 0.33$), there were no significant effects (all other *p*s > .117 and all $d_z < 0.21$). ANOVAs with the factors of measure (i.e., SOA vs. RT) and mapping (task-to-hand vs. task-to-finger) separately for each block part revealed no significant interaction for the first ($p = .200$; $\eta_p^2 = .05$) and second part ($p = .178$; $\eta_p^2 = .06$).

Summary. The analyses involving the within-subject factor part within a mapping condition (first part of blocks vs. second part of blocks) further demonstrates that switching behavior generally increases over time by showing higher switch rates for the second compared to first part *within* each task mapping condition. More importantly, these analyses also demonstrate that substantial practice is not needed to adapt to the task mapping manipulation. Specifically, there were higher switch rates in the task-to-finger compared to the task-to-hand condition independent from whether the first or second part of blocks of the specific-mapping condition were compared.

Appendix C (Experiment 2)

In this appendix, we present complementary analyses to explore whether and how our central task mapping manipulation has influenced task performance in the forced choice trials.

Task performance in forced-choice trials (exploratory analyses): Reaction times (RT) and percentage errors (PE). As for the free choice task performance analyses, The RT-ANOVA with the within-subject factors) of transition (repetition vs. switch) and mapping (task-to-finger vs. task-to-hand) revealed a significant main effect of transition (i.e., switch costs), $F(1, 69) = 442.45$, $p < .001$, $\eta_p^2 = .87$, and a significant interaction, $F(1, 69) = 33.64$, $p < .001$, $\eta_p^2 = .33$ (with $p = .862$, $\eta_p^2 < .01$ for the main effect of mapping). Switch costs were larger in the task-to-hand (807–547=260 ms) compared to task-to-finger condition (781–575=206 ms). The PE-ANOVA only yielded a significant main effect of transition reflecting overall switch costs of 5.99–2.04=3.95%, $F(1, 69) = 474.41$, $p < .001$, $\eta_p^2 = .87$ (with all other *p*s > .259, $\eta_p^2 < .03$).

Appendix D (Experiment 2 and 3)

In this appendix, we provide a direct comparison of switch rates between Experiment 2 and Experiment 3.

Comparison of switch rates between Experiment 2 and Experiment 3: A mixed ANOVA with the within-subject factor mapping and the between-subject factor experiment revealed a significant main effect of experiment, $F(1, 120) = 61.22$, $p < .001$, $\eta_p^2 = .34$, reflecting larger switch rates in Experiment 3 (.40) than in Experiment 2 (.21). There was also a significant main effect of mapping, $F(1, 120) = 15.17$, $p < .001$, $\eta_p^2 = .11$, reflecting larger switch rates with task-to-finger (.31) than task-to-hand (.27) mapping. Critically, the interaction was also significant, $F(1, 120) = 61.22$, $p = .006$, $\eta_p^2 = .06$.

Figure A1. Cumulative distribution functions (CDF) of switch SOAs separately for the task-to-hand and task-to-finger condition.

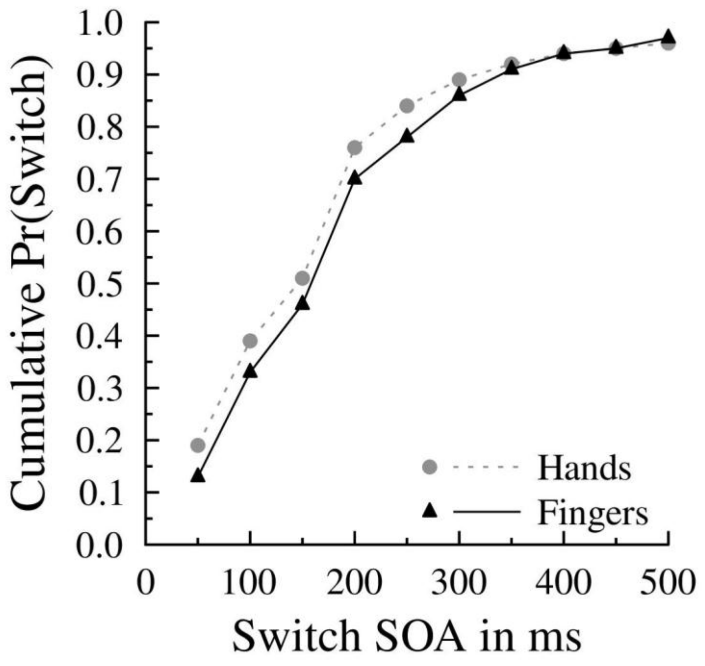

Figure A2. A. Scatter plots of individual median switch costs against individual switch rates separately for the task-to-hand and task-to-finger condition. B. Scatter plots of individual median switch costs against individual median switch SOA separately for the task-to-hand and task-to-finger condition.

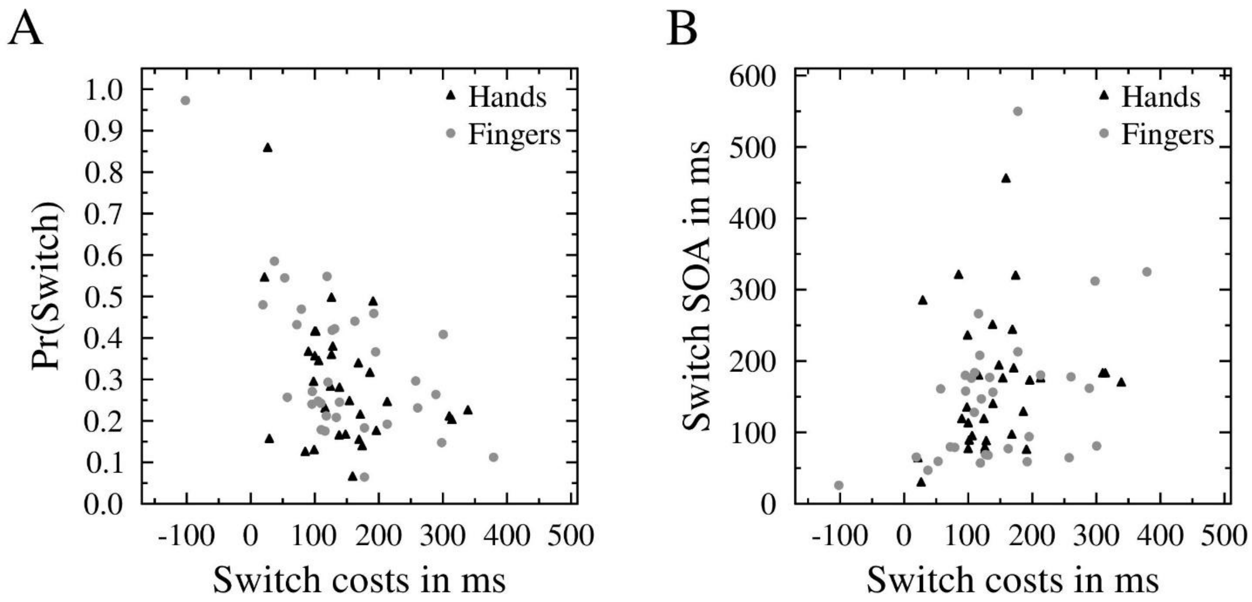

Table B1

Mean switch rates, mean median reaction time (RT) and mean percentage errors (PE) as a function of trial transition (i.e., task switch vs. task repetition), mean median RT switch costs and mean PE switch costs (i.e., task switch RT/PE–task repetition RT/PE) and mean median switch stimulus-onset-asynchrony (SOA separately for the two task mapping condition and as a function of task mapping order and first and second half of the condition-specific blocks. Standard error of the means in parentheses.

	Task mapping order			
	Hand-then-Finger blocks order (*N* = 16)		Finger-then-Hand blocks order (*N* = 16)	
Measure	Hands	Fingers	Hands	Fingers
Switch rate	.27 (.05)	.37 (.05)	.32 (.03)	.30 (.03)
Switch SOA	183 (26)	144 (31)	145 (19)	156 (22)
Task switch RT	597 (24)	559 (22)	558 (16)	578 (21)
Task repetition RT	437 (10)	429 (14)	428 (9)	419 (12)
Switch costs RT	160 (22)	130 (26)	131 (13)	159 (23)

	Block part			
	First part (*N* = 32)		Second part (*N* = 32)	
Measure	Hands	Fingers	Hands	Fingers
Switch rate	.27 (.03)	.31 (.05)	.31 (.03)	.35 (.03)
Switch SOA	174 (17)	155 (20)	162 (18)	148 (18)
Task switch RT	614 (18)	611 (22)	554 (14)	553 (14)
Task repetition RT	435 (7)	424 (9)	462 (36)	424 (10)
Switch costs RT	178 (18)	188 (24)	92 (38)	129 (23)
